# Margin assessment during breast conserving surgery using diffuse reflectance spectroscopy

**DOI:** 10.1117/1.JBO.29.4.045006

**Published:** 2024-04-25

**Authors:** Dinusha Veluponnar, Lisanne L. de Boer, Behdad Dashtbozorg, Lynn-Jade S. Jong, Freija Geldof, Marcos Da Silva Guimaraes, Henricus J. C. M. Sterenborg, Marie-Jeanne T. F. D. Vrancken-Peeters, Frederieke van Duijnhoven, Theo Ruers

**Affiliations:** a Netherlands Cancer Institute, Antoni van Leeuwenhoek, Department of Surgery, Image-Guided Surgery, Amsterdam, The Netherlands; b University of Twente, Department of Nanobiophysics, Faculty of Science and Technology, Enschede, The Netherlands; c Netherlands Cancer Institute, Antoni van Leeuwenhoek, Department of Pathology, Amsterdam, The Netherlands; d Amsterdam University Medical Center, Department of Biomedical Engineering and Physics, Amsterdam, The Netherlands

**Keywords:** diffuse reflectance spectroscopy, resection margin assessment, breast-conserving surgery, tissue classification

## Abstract

**Significance:**

During breast-conserving surgeries, it is essential to evaluate the resection margins (edges of breast specimen) to determine whether the tumor has been removed completely. In current surgical practice, there are no methods available to aid in accurate real-time margin evaluation.

**Aim:**

In this study, we investigated the diagnostic accuracy of diffuse reflectance spectroscopy (DRS) combined with tissue classification models in discriminating tumorous tissue from healthy tissue up to 2 mm in depth on the actual resection margin of *in vivo* breast tissue.

**Approach:**

We collected an extensive dataset of DRS measurements on *ex vivo* breast tissue and *in vivo* breast tissue, which we used to develop different classification models for tissue classification. Next, these models were used *in vivo* to evaluate the performance of DRS for tissue discrimination during breast conserving surgery. We investigated which training strategy yielded optimum results for the classification model with the highest performance.

**Results:**

We achieved a Matthews correlation coefficient of 0.76, a sensitivity of 96.7% (95% CI 95.6% to 98.2%), a specificity of 90.6% (95% CI 86.3% to 97.9%) and an area under the curve of 0.98 by training the optimum model on a combination of *ex vivo* and *in vivo* DRS data.

**Conclusions:**

DRS allows real-time margin assessment with a high sensitivity and specificity during breast-conserving surgeries.

## 1 Introduction

Breast cancer is the most frequently diagnosed type of cancer among women globally, with an estimated incidence of 2.26 million cases in the year 2020.^1^ It is the leading cause of cancer death among females, with an estimated number of 685,000 deaths in the year 2020.^1^ The standard of care for early-stage breast cancer is breast-conserving therapy (BCT), which involves breast-conserving surgery (BCS) followed by adjuvant radiotherapy.^2^^,^^3^ In BCS, the surgeon attempts to excise the entire tumor along with a small margin of healthy tissue while providing a satisfactory cosmetic outcome. Multiple randomized clinical trials have demonstrated equivalent survival outcomes for mastectomy and BCT if clear resection margins (edges of a breast cancer specimen) are achieved during BCS.^4^^–^^7^ To assess whether the margins of a specimen are clear, and therefore no cancerous cells remain in the adjacent tissue, a histopathological examination is performed after surgery. If positive (tumor-involved) margins are found, the patient is usually recommended to undergo a re-excision and/or additional doses of radiotherapy,^8^ as tumor positive margins are associated with a twofold increase in ipsilateral breast tumor recurrence and an overall lower long-term survival.^9^^–^^13^ The required additional therapy in patients with positive margins may result in anxiety, increased clinical complications,^14^^,^^15^ unsatisfactory cosmetic outcome, and^16^^–^^19^ increased health care costs.^15^^,^^20^^–^^22^ Globally, there is a lack of consensus on what constitutes a “positive margin,” and the reported rates of positive margins after BCS in the literature vary from 9% to as high as 36% for invasive breast cancer and from 4% to as high as 23% for ductal carcinoma *in situ* (DCIS).^23^ According to Dutch guidelines, a positive margin is defined as invasive carcinoma (IC) cells reaching the inked margin over a trajectory >4  mm or DCIS cells reaching the inked margin over any trajectory.^24^ Focally positive margins are defined as IC cells reaching the inked margin over a trajectory ≤4  mm.^24^ Otherwise, the margins are considered negative. For the United States, according to the guidelines of the Society of Surgical Oncology (SSO) and the American Society for Radiation Oncology (ASTRO), a margin is considered tumor-positive when ink touches the IC or when there is DCIS present within 2 mm from the resection margin.^9^

During BCS, the surgeon has to rely on visual and haptic inspection of the tissue to obtain information about the surgical margins. The definitive margin status is only reported following the histopathological examination, which usually occurs a few days after surgery. Therefore, there is a clinical need for methods that are able to support the surgeon’s evaluation of resection margins in real time and accurately during BCS. Current techniques for intraoperative margin assessment involve specimen radiography, frozen section analysis, and touch preparation cytology. However, these techniques are not incorporated into the standard surgical workflow due to low diagnostic accuracy, low speed, and cumbersome workload for pathologists and/or radiologists.^25^^,^^26^ In light of this, a wide variety of approaches with the overarching goal of intraoperative margin assessment is being investigated.^27^^–^^29^ These techniques include intraoperative ultrasonography,^30^^–^^32^ radiofrequency spectroscopy,^33^^–^^36^ bioimpedance spectroscopy,^37^ Raman spectroscopy,^38^^–^^40^ digital breast tomosynthesis,^41^ fluorescence imaging,^42^ microcomputed tomography,^43^ optical coherence tomography,^44^^–^^46^ quantitative micro-elastography,^47^ ultraviolet photoacoustic microscopy,^48^^,^^49^ intraoperative flow cytometry,^50^ and microscopy with ultraviolet surface excitation.^51^ Although some of the mentioned techniques seem promising, none of them have been translated into routine surgical practice yet. The reasons for this lack of implementation include low diagnostic accuracy,^41^^,^^43^^,^^52^ time-consuming process, difficult interpretation, high operator dependence, inability to perform over the entire margin,^50^ early stage of development, and/or undetermined cost-effectiveness.^27^^,^^28^^,^^53^^,^^54^

Fiber-optic diffuse reflectance spectroscopy (DRS) is an optical technique that is also being investigated for the purpose of margin assessment in cancer surgeries. In DRS, a beam of light is directed through an emitting fiber into the tissue, where it undergoes several light-tissue interactions, such as scattering and absorption, before the light is partially reflected back. The intensity of the diffusely reflected light at various wavelengths is recorded, producing a diffuse reflectance spectrum. By analyzing this spectrum, information can be obtained about the composition and optical properties of the illuminated tissue. This information could be used to distinguish different tissue types and thus potentially detect cancerous tissue during cancer surgeries.

In recent years, our research group has conducted multiple studies to investigate the combination of DRS with classification models to distinguish tumorous tissue from healthy tissue on sliced, *ex vivo* breast cancer specimens with excellent results, including a sensitivity and specificity of 100% in distinguishing tumorous breast tissue from healthy breast tissue.^55^^,^^56^ In these studies, DRS measurements were performed on pure healthy tissue locations and pure tumorous tissue locations on sliced breast cancer specimens. In our latest publication, we reported the results of our study on the diagnostic accuracy of DRS combined with classification models, as a margin assessment tool during BCS. We applied DRS on the actual resection margin of *ex vivo* breast cancer specimens rather than sliced specimens and found a sensitivity of 93% and a specificity of 75% for distinguishing tumorous tissue from healthy tissue up to 2 mm from the margin, when using five optical fibers.^57^

The next step is to move forward to real-time clinical application during breast-conserving surgeries and investigate if these classification models that are trained and tested on DRS data acquired from *ex vivo* breast tissue have a similar performance when applied to DRS data from *in vivo* breast tissue. This is relevant because acquiring data *in vivo* is more complex as it is time-consuming and usually limited to a small number of measurements per patient. Therefore, it is worthwhile to investigate if one could mainly use *ex vivo* data for training a classification model without compromising the performance when used *in vivo*.

The first aim was to investigate the performance of DRS combined with classification algorithms, trained on DRS data acquired from *ex vivo* and/or *in vivo* breast tissue, and tested on data acquired from *in vivo* breast tissue. For this, we acquired an extensive dataset of DRS data from *ex vivo* and *in vivo* breast tissue. We built four different types of classification models, and evaluated their classification performance when trained on the different types of data (*ex vivo*/*in vivo*). The second aim was to select the optimum model based on the highest classification performance and investigate the model training strategy that yields the best performance for the selected classification model.

## 2 Materials and Methods

### 2.1 Diffuse Reflectance Spectroscopy Setup

The instrumentation of the optical measurement system consisted of a console with five light sources for tissue illumination, two different spectrometers, and an in-house developed, handheld fiber-optic DRS probe. The light sources were identical, halogen broadband lamps (Avantes, AvaLight-HAL, 360 to 2500 nm) with integrated shutters. Of both spectrometers, one covered a visible wavelength range from 200 to 1160 nm (Avantes, AVASPEC-HS2048XL-EVO), and the other covered a near-infrared wavelength range from 900 to 1750 nm (Avantes, AVASPECNIR256-1.7-RS). The DRS probe consisted of five peripheral illumination fibers placed in a circle around one receiving fiber in the center. Each light source was coupled to one illumination fiber. The source–detector fiber distance for all fibers was 2.0 mm. All diffuse reflectance spectra were calibrated on a white reference object of spectralon in a similar manner as described in Refs. 56 and 58. Therefore, any disparity between the light sources would be inconsequential. A schematic representation of the optical measurement system is given in Sec. 1 in the Supplementary Material.

### 2.2 Study Design and Participants

This prospective non-randomized cohort study was conducted from 2019 to 2023 at the Netherlands Cancer Institute - Antoni van Leeuwenhoek Hospital (NKI-AvL). Ethical approval for the study protocol was granted by the Institutional Review Board. Patients undergoing breast-conserving surgery due to histologically proven IC and/or DCIS, with or without neoadjuvant therapy, were included. Patients with a complete radiologic response after neoadjuvant therapy were excluded. The study period consisted of two phases. During the first phase, patients were included for only *ex vivo* tissue measurements on BCS specimens. According to the medical research involving human subjects act, no written consent was required for these measurements. During the second phase of the study, patients were included for *in vivo* as well as *ex vivo* tissue measurements during BCS. All included participants for these measurements had provided written informed consent and were treated according to local standard protocols.

### 2.3 Data Acquisition

**Fig. 1:**
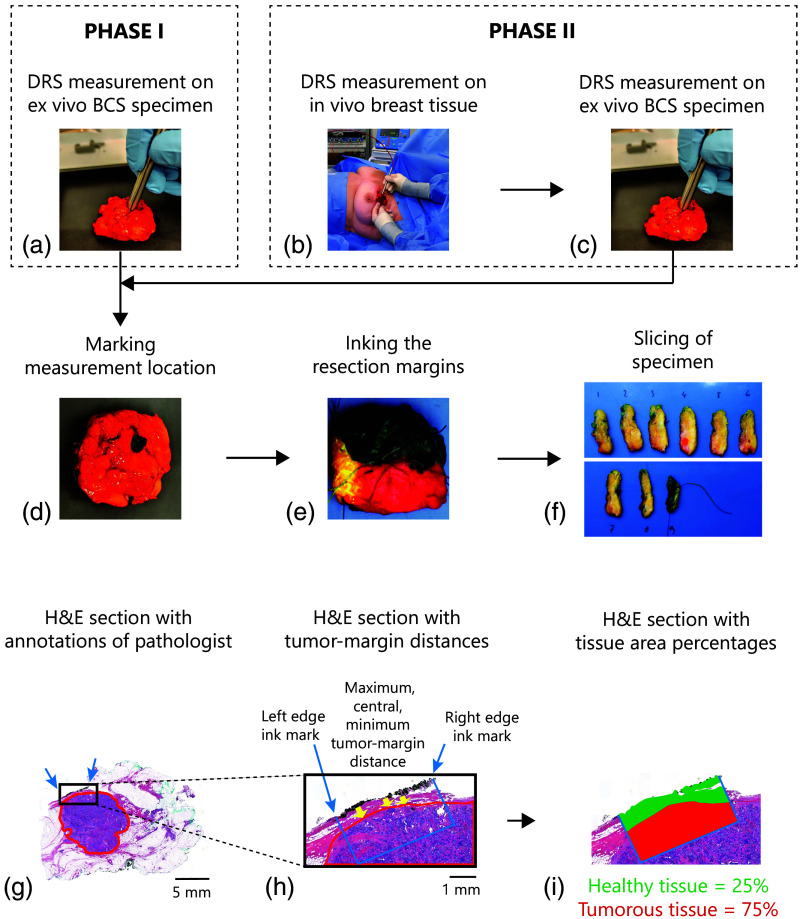
Overview of the data acquisition method for phase I (top left) and phase II (top right). In panel (a), the image displays a point-based DRS measurement on an *ex vivo* BCS specimen. This is followed by (d) (middle row) marking the measurement location with black pathology ink, (e) standard processing by the pathology department, including coloring, and (f) slicing the specimen. In phase II, the workflow starts with (b) point-based DRS measurements on a volume *in vivo* breast tissue that is meant to be resected, followed by (c) DRS measurement on an *ex vivo* BCS specimen. This is followed by the same steps (d)–(f) as explained for phase I. A schematic overview of the method for determining the tumor-margin distance and tissue area percentages in the corresponding H&E sections (bottom). In panel (g), the original H&E section with the annotated borders of the lesion in red and the black ink along the margin at the measurement location (blue arrows) and (h) the magnified image of the measured tissue location (blue arrows). The yellow arrows indicate the maximum, central, and minimum tumor-margin distance, determined by measuring the perpendicular distance from the surgical surface to the tumor. Finally, the percentage of tumorous tissue and healthy breast tissue was determined over a depth of 2 mm at the marked region (i), indicated by the blue box (h).

A schematic representation of the data acquisition process is shown in Fig. 1. The following patient characteristics were collected from medical records: age at the time of surgery, administration of neoadjuvant therapy, pathological diagnosis of the resected specimen, and margin status.

#### 2.3.1 Measurements phase I

Directly after surgery, the BCS specimen was collected from the surgical team, and DRS measurements were performed after an estimated time interval of a few minutes. We selected approximately three to five locations on the margins of the specimen for DRS measurements. A total of three consecutive DRS measurements were performed on each selected location while ensuring the surface of the probe tip was in contact with the tissue. After the measurements, the measured tissue location was marked with black pathology ink. Considering the overall limited number of measurements that were possible due to the histopathology protocol in our hospital, we performed ultrasound imaging on some of the specimens to localize the areas with a short distance from the tumor edge to the resection margin.

#### 2.3.2 Measurements phase II

During this phase, *in vivo* measurements as well as *ex vivo* measurements were performed. Prior to the surgery, the optical measurement system was set up. During the standard surgical procedure, optical measurements were obtained by the surgeon at one location on each of the six resection sides of the tissue volume meant to be resected (comparable to six sides of a cube). Each location was selected based on a suspected short distance between the margin and the tumor edge after visual and haptic inspection by the surgeon. Similar to the *ex vivo* measurements, three consecutive DRS spectra were acquired on each location. This was followed by the placement of a surgical clip to mark it. The acquisition time of each measurement was a few seconds. After surgery, we removed each surgical clip on the *in vivo* measurement locations and directly marked it again with black pathology ink. Furthermore, we performed DRS measurements on one or two additionally selected locations as per the measurement protocol of phase I.

#### 2.3.3 Specimen handling and ground truth labels

After DRS measurements, the specimen was delivered to the pathology department for standard pathology processing. This processing entailed inking the resection margins for orientation purposes, freezing the specimen, slicing the specimen in a serial manner, and processing the slices into cellular thin hematoxylin and eosin-stained (H&E) sections. The digitized pathological H&E sections of all measurement locations were examined by an experienced pathologist. During this process, the pathologist annotated all areas of IC and DCIS in the H&E images. Subsequently, a region up to 2 mm underneath the black ink mark was determined. The area percentage of tumor tissue within this region was determined based on the annotations, using image processing tools in MATLAB. The remaining healthy tissue in the region consisted of fat and connective tissue. The area percentages of both healthy tissue types were determined using a threshold value for the intensity in the green channel of the H&E image, as described in Kho et al.^59^ These tissue percentages together form the ground truth labels of the dataset. If any tumor tissue was present within the determined region up to a depth of 2 mm, the location was labeled as malignant. The rationale behind choosing this depth is the definition of a positive margin according to the SSO ASTRO guidelines mentioned earlier.^9^ If no tumor tissue was present in the region, the location was labeled as healthy. Additionally, if any tumor percentage was present within the region, the maximum, minimum, and central perpendicular distance from the black ink to the tumor edge was determined. The tumor-margin distance was calculated as the average of the aforementioned distances.

### 2.4 Data Processing

**Fig. 2:**
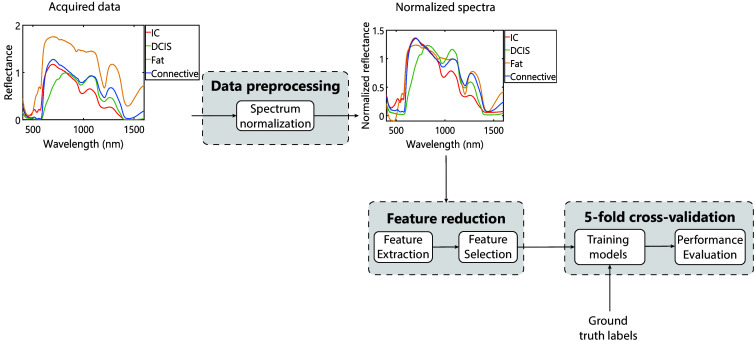
Overview of the workflow for data analysis. The process started with data preprocessing, which entailed spectrum normalization. The plotted spectra on the left are examples of characteristic spectra from locations with IC, DCIS, and fat, respectively, connective tissue. The plots on the right are the same spectra after normalization was applied. The next step was feature reduction, which involved feature extraction and feature selection. Subsequently, the selected features coupled with the ground truth labels derived from the histopathological analysis were used to train different classification models using fivefold cross-validation. The performance of each model was evaluated.

For all data analyses, we used MATLAB version R2022a (MathWorks Inc., Natick, Massachusetts, United States). The entire data analysis process is displayed in Fig. 2.

#### 2.4.1 Spectra normalization

The three consecutively collected diffuse reflectance spectra of each measurement location were averaged, and the spectra from both wavelength ranges were stitched together. Moreover, the extremities of the spectral range were removed due to a large level of noise relative to the signal being measured. These extremities consisted of the spectral range between 350 and 400 nm and the spectral range between 1600 and 1700 nm. As a result, the remaining data were of higher quality and more reliable for further processing. A multiplicative scatter correction (MSC) normalization approach was applied^60^ to correct the spectra in a manner that they are as close as possible to the mean spectrum of the dataset. By applying this technique to the dataset, intensity differences due to variability in light scattering effects and path length were corrected. Furthermore, random inter-patient variations and random variations in background light and signal quality were also reasonably reduced.

#### 2.4.2 Feature extraction and selection

Each normalized spectrum consisted of the reflection intensities at the wavelength range 400 to 1600 nm, meaning 1200 different wavelengths. Each reflection intensity represents a feature that could be used for training classification models. To reduce overfitting and improve classification performance, we performed feature extraction and selection as described in de Boer et al.^56^ The extracted features were a set of visible spectral features in the near-infrared wavelength range, including the slopes of spectra between the maximum intensities in the first 25% and the last 25% of particular wavelength ranges, the maximum difference between the slope and the spectrum, the corresponding wavelength at the point of maximum difference, and the inflection points left and right of the point of maximum difference, as described by de Boer et al.^56^ This set included 80 different features over the spectral range that we measured. Because we measured using 5 different fibers, in total 400 features could be extracted (80 features per fiber). This was followed by feature selection, which entailed applying a minimum redundancy maximum relevance (MRMR) feature selection algorithm.^61^ MRMR is used to rank the importance of all quantified features for the binary classification of each spectrum into belonging to either tumorous tissue or healthy tissue. Features with an importance score ≥0.015 were selected.

### 2.5 Classification

Four different types of supervised machine learning classification models were built: (1) linear support vector machine (SVM); (2) quadratic SVM, (3) weighted K-nearest neighbors (KNNs) algorithm, and (4) ensemble random under sampling boosted tree (RUSBoost). We selected these particular model types because our research group has built similar models for DRS data classification during earlier studies, which showed high classification performance.^56^^,^^57^^,^^62^^–^^64^ Three classification models per model type were built, each trained on one of the following types of data: (1) spectral features from *ex vivo* tissue locations, (2) spectral features from *in vivo* tissue locations, and (3) spectral features from *ex vivo* and *in vivo* tissue locations. Then, each classification model was tested on spectral features from *in vivo* tissue locations. For each experiment, the data were divided into training and test sets using a repeated 5-fold cross-validation method, with 20 iterations. The data from each patient were assigned to either the training set or the test set during each iteration to avoid bias.

### 2.6 Performance Evaluation

First, we evaluated the performance of each classification model using the following metrics, averaged over 20 iterations: Matthews correlation coefficient (MCC) with corresponding standard deviation (SD). The MCC ranges between −1 and 1 and indicates the agreement between the actual and predicted labels. This metric considers the true negative ($N^{+}$) rate, the true positive ($P^{+}$) rate, the false negative ($N^{-}$) rate, and the false positive ($P^{-}$) rate and is calculated as follows:

$$\mathrm{MCC}=\frac{\left(P^{+}\times N^{+}-P^{-}\times N^{-}\right)}{\sqrt{\left(P^{+}+P^{-})(P^{+}+N^{-})(N^{+}+P^{-})(N^{+}+N^{-}\right)}}.$$ (1)

It is a robust measure that only produces a high score if the prediction has good rates in all four categories. This metric takes imbalance between classes into account. We compared the MCC of the different classification models, when trained on the three different types of data. Subsequently, we selected the model type with the overall highest MCC for further analysis. We plotted receiver operating characteristic (ROC) curves of the selected models trained on the three different types of data. Subsequently, we selected the optimal operating point for each ROC curve, which was defined as the point with the minimum average misclassification cost. The sensitivity and specificity with the corresponding 95% confidence intervals (CI) at each of these points were determined. The threshold for calculating sensitivity and specificity was selected based on the highest MCC value. Finally, we plotted the tumor-margin distance against the tumor percentage of all correctly and incorrectly classified tumor locations. We investigated the correlation between these parameters and the misclassification of tumor locations using the aforementioned plot.

## 3 Results

### 3.1 Characteristics of Patient Cohort

A total of 228 patients were recruited into this study. An overview of the patient and tumor characteristics as well as measurement locations of all included patients in phase I (N=128) and phase II (N=100) are given in Table 1. The median age of the patients in phase I was 60.5 years (SD = 11.9) compared with 62.0 years (SD = 13.1) in phase II (Table 1). The median lesion diameter was 13.5 mm (SD = 12.6) in phase I compared with 14.0 (SD = 13.5) in phase II (Table 1). Both the median age and the median lesion diameter were comparable in both groups. Regarding neoadjuvant treatment (chemotherapy or endocrine therapy), both patient groups had a comparable rate of patients without neoadjuvant treatment (63.3% of patients in phase I compared with 62% of patients in phase II) (Table 1). In phase I, a slightly higher percentage of patients had received neoadjuvant chemotherapy (27.3%) compared with phase II (22%) (Table 1). In phase II, a slightly higher percentage of patients had received neoadjuvant endocrine therapy (16%) compared with phase I (9.4%) (Table 1). It should be mentioned that many different (combinations of) malignant pathological diagnoses have been included in both phases and the distribution was quite comparable, as can be seen in Table 1. The majority of all patients in phase I (42,2%) and phase II (39%) had an IC NST (IC of no special type). This was followed by IC NST combined with DCIS (28.1% in phase I and 36% in phase II) (Table 1). When looking at margin status, according to Dutch guidelines, 14,0% of patients in phase I had positive margins compared with 12% of patients in phase II (Table 1). In total, patients in phase I had a slightly higher rate of patients with focally positive margins (18%) compared with patients in phase II (10%) (Table 1).

**Table 1:** Characteristics of patient cohort and measurement locations.

	Phase I: *Ex vivo* (N=128)	Phase II: *In vivo* and *ex vivo* (N=100)
**Age** (years) (median, SD)	60,5 (11,9)	62,0 (13,1)
**Lesion diameter** (mm) (median, SD)	13,5 (12,6)	14,0 (13,5)
**Neoadjuvant treatment**		
Chemotherapy	35 (27,3%)	22 (22%)
Endocrine therapy	12 (9,4%)	16 (16%)
None	81 (63,3%)	62 (62%)
**Histological tumor type**		
IC NST	54 (42,2%)	39 (39%)
IC NST + DCIS	36 (28,1%)	36 (36%)
IC NST + LCIS	4 (3,1%)	1 (1%)
ILC	13 (10,2%)	11 (11%)
ILC + LCIS	6 (4,7%)	2 (2%)
ILC + DCIS	1 (0.8%)	0
DCIS	13 (10,1%)	8 (8%)
LCIS	1 (0.8%)	2 (2%)
DCIS + LCIS	0	1 (1%)
**Margin status**		
Negative	87 (68,0%)	78 (78%)
Focally positive	23 (18,0%)	10 (10%)
Positive	18 (14,0%)	12 (12%)
**Measurement locations**		
Tumor locations *in vivo*	—	31
Tumor locations *ex vivo*	106	46
Healthy locations *in vivo*	—	483
Healthy locations *ex vivo*	447	80

IC NST, invasive carcinoma of no special type; DCIS, ductal carcinoma *in situ*; ILC, invasive lobular carcinoma; and LCIS, lobular carcinoma in situ.

### 3.2 Measurement Locations

Of all 553 measurement locations acquired in phase I, there were 106 (19.2%) *ex vivo* tumor locations (tumor tissue within 2 mm from the margin) and 447 (80.8%) *ex vivo* healthy locations (healthy tissue within 2 mm from the margin) (Table 1). Among the 640 measurement locations acquired in phase II, there were 46 (7.2%) *ex vivo* tumor locations, 31 (4.8%) *in vivo* tumor locations, 80 (12.5%) *ex vivo* healthy locations, and 483 (75.5%) *in vivo* healthy locations (Table 1).

**Fig. 3:**
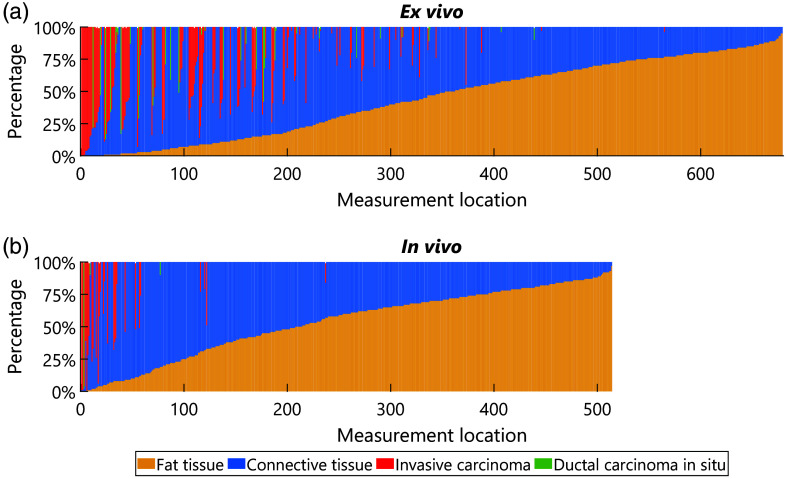
(a) Distribution of area percentages per tissue type for all *ex vivo* measurement locations. (b) Distribution of area percentages per tissue type for all *in vivo* measurement locations.

Figure 3 displays the distribution of the area percentages of all tissue types in the correlated H&E region of each *ex vivo* measurement location (top) and each *in vivo* measurement location (bottom). The tissue type distributions are quite similar for both groups, except for four notable differences. The first difference is that the percentage of measurement locations with tumor tissue (either IC or DCIS) is higher in the *ex vivo* group (22%) compared with the *in vivo* group (6%) (Fig. 3). The second difference is that the average percentage of tumor tissue in the tumor locations is higher among the *in vivo* locations compared with the *ex vivo* locations (Fig. 3). The third difference is that a higher percentage of tumor locations in the *ex vivo* group contain DCIS (22%) compared with the tumor locations in the *in vivo* group (10%) (Fig. 3). The fourth difference is that the locations in the *in vivo* group on average have a slightly higher area percentage of fat tissue compared with the *ex vivo* group (Fig. 3).

### 3.3 Selected Features

The MRMR analysis yielded 21 optimum features per spectrum. Table 2 gives an overview of all selected optimum features, ranked by feature importance score. It is important to mention that all of the described features are in the near-infrared wavelength range. In this particular range, fat, water, and collagen are the primary absorbers in tissue that affect the interaction with light.

**Table 2:** Optimum features according to MRMR analysis, ranked by feature importance score.

Rank	Selected feature
1	Slope of the measured spectrum between 1213 and 1248 nm
2	Slope of the measured spectrum between 931 and 1195 nm
3	Wavelength of the left inflection point of the peak between 1224 and 1331 nm
4	Maximum difference between the slope and the measured spectrum between 1142 and 1225 nm
5	Maximum difference between the slope and the measured spectrum between 1021 and 1102 nm
6	Slope of the measured spectrum between 999 and 1034 nm
7	Slope of the measured spectrum between 1395 and 1430 nm
8	Wavelength of the right inflection point of the peak between 1404 and 1437 nm
9	Slope of the measured spectrum between 1467 and 1502 nm
10	Wavelength of the right inflection point of the peak between 1142 and 1225 nm
11	Slope of the measured spectrum between 1121 and 1401 nm
12	Slope of the measured spectrum between 932 and 967 nm
13	Slope of the measured spectrum between 1112 and 1147 nm
14	Wavelength of the right inflection point of the peak between 1224 and 1331 nm
15	Slope of the measured spectrum between 1201 and 1236 nm
16	Wavelength of the right inflection point of the peak between 1382 and 1574 nm
17	Wavelength of the right inflection point of the peak between 1404 and 1437 nm
18	Slope of the measured spectrum between 883 and 1149 nm
19	Maximum difference between the slope and the measured spectrum between 1224 and 1329 nm
20	Slope of the measured spectrum between 932 and 967 nm
21	Slope of the measured spectrum between 926 and 1149 nm

### 3.4 Classification Performance of Four Types of Classification Models Trained on Different Types of Data

Table 3 gives an overview of the MCCs for the discrimination of tumor locations from healthy locations for four types of classification models, trained on three different types of data and tested on the *in vivo* dataset. From this table, it is apparent that training on DRS data acquired from *ex vivo* tissue yields similar performance results compared with training on DRS data acquired from *in vivo* tissue, regardless of model type. Furthermore, it is noticeable that the RUSBoost has the overall best classification performance on any type of training data compared with the other model types (Table 3). Overall, the highest classification performance is achieved by the RUSBoost trained on a combination of *in vivo* and *ex vivo* data, with an MCC of 0.76 (Table 3). The quadratic SVM and weighted KNN have the lowest performance with an MCC ranging between 0.57 and 0.59 and 0.58 and 0.61, respectively, depending on the type of training data (Table 3). For the remainder of the study, we selected the RUSBoost as the optimum model type for further analysis.

**Table 3:** Classification performance for four different types of classification models, when trained on different types of DRS data and tested on *in vivo* data.

	Type of training data	Linear SVM	Quadratic SVM	Weighted KNN	RUSBoost
MCC (SD)	*Ex vivo*	0.67 (0.007)	0.57 (0.016)	0.59 (0.007)	0.73 (0.010)
	*In vivo*	0.62 (0.055)	0.58 (0.048)	0.58 (0.036)	0.74 (0.029)
	*In vivo + Ex vivo*	0.71 (0.037)	0.59 (0.055)	0.61 (0.021)	0.76 (0.028)

### 3.5 Effect of Different Types of Training Data on Classification Performance of Optimum Model Type

**Fig. 4:**
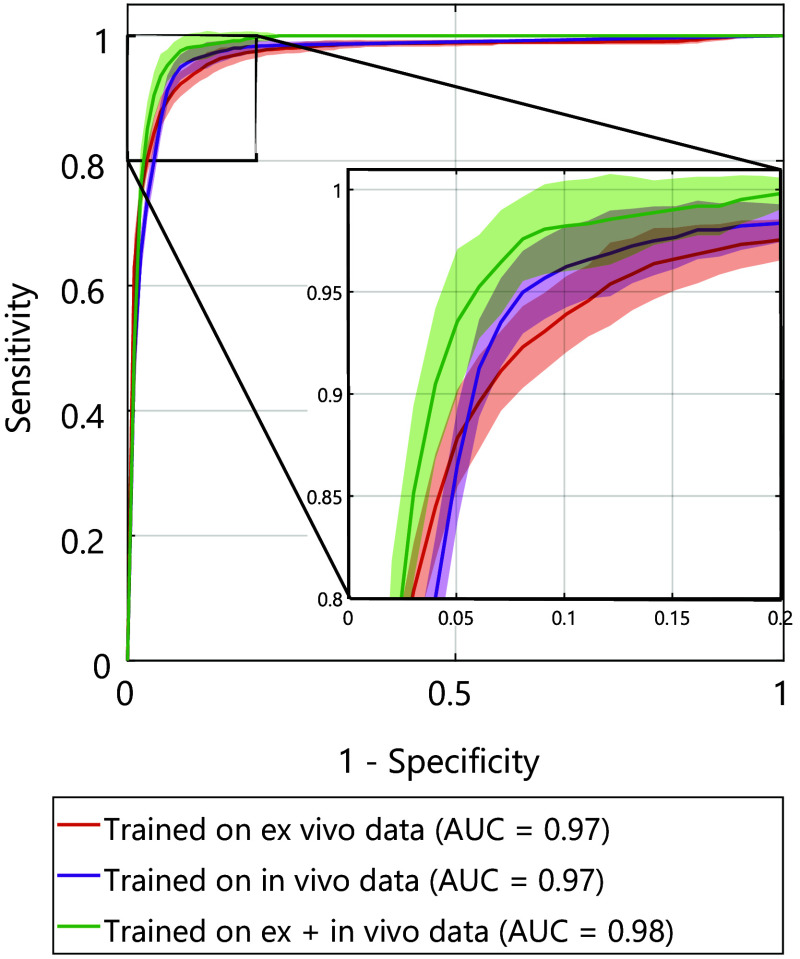
ROC curves for RUSBoost model when trained on different types of data.

Figure 4 shows the ROC curves and the area under the curve (AUC) for the RUSBoost, when trained on different types of training data. It is noteworthy to mention that the AUC of all differently trained models is similarly high, indicating a high classification performance (Fig. 4).

Table 4 displays the sensitivity and specificity with the 95% CI at the optimal operating point of each of the respective ROC curves. Training on a combination of *ex vivo* and *in vivo* data yields a slightly higher sensitivity (96.7% (95% CI 95.6% to 98.2%) and specificity (90.6% (95% CI 86.3% to 97.9%) compared with models trained on either *ex vivo* or *in vivo* data. This finding is in accordance with the marginally higher MCC (Table 3) and AUC (Fig. 4) of this model.

**Table 4:** Sensitivity and specificity of the RUSBoost model for distinguishing tumorous tissue from healthy breast tissue *in vivo*, when trained on different types of data.

	Sensitivity (%) (95% CI)	Specificity (%) (95% CI)
**Trained on** ***in vivo*** **data**	96.1 (94.6 to 97.9)	87.6 (70.2 to 93.4)
**Trained on** ***ex vivo*** **data**	96.0 (94.5 to 97.3)	85.7 (77.1 to 89.2)
**Trained on** ***ex vivo*** **and** ***in vivo*** **data**	96.7 (95.6 to 98.2)	90.6 (86.3 to 97.9)

### 3.6 Classifiction of Tumor Locations *In Vivo*

**Fig. 5:**
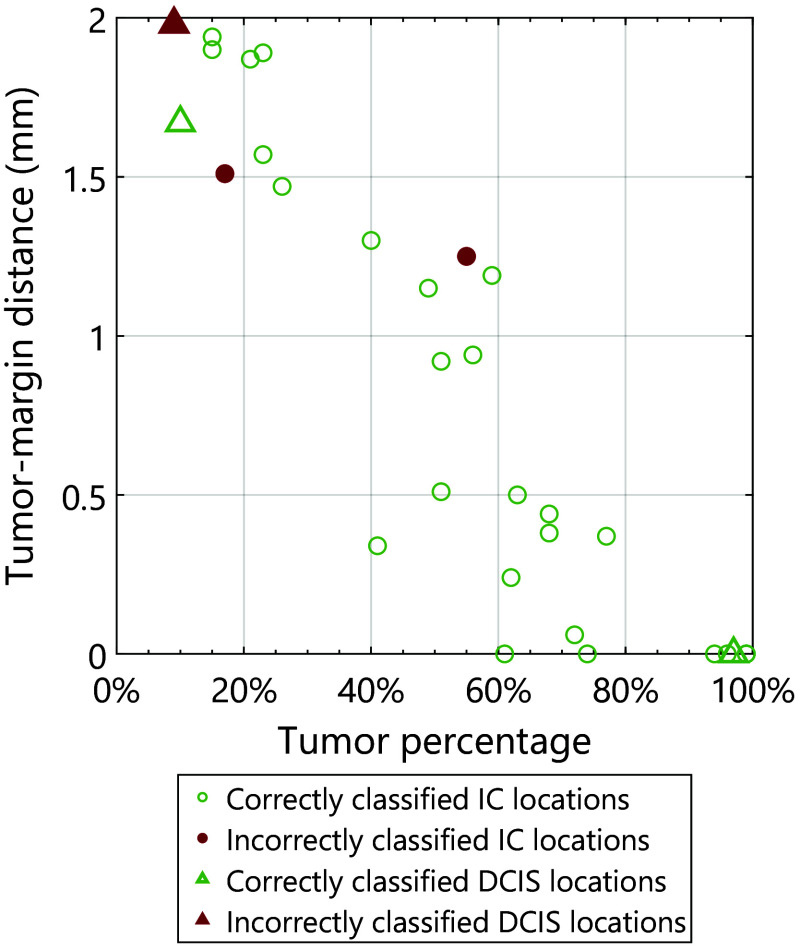
Tumor-margin distance compared with tumor area percentage of all correctly and incorrectly classified IC and DCIS locations *in vivo*.

Figure 5 displays the correctly and incorrectly classified tumor locations (IC and DCIS) with their corresponding tumor-margin distance and area percentage of tumor for the *in vivo* measurements. Overall, there appears to be a negative, linear correlation between tumor percentage and the distance to the tumor margin. This was found in our earlier research as well.^65^ This occurs because the tumor percentage for each measurement location is evaluated within a two-dimensional region extending up to 2 mm from the margin. When the average distance to the tumor is greater, it is likely that there will be a lower percentage of tumor tissue in that area. In total, there were 31 *in vivo* tumor locations in the entire dataset (Table 1). As can be seen in Fig. 5, most of the tumor locations (28) were identified correctly, and only 3 tumor locations were misclassified. All misclassified locations have a distance >1.0  mm from the resection margin, which according to the Dutch guidelines is not considered a positive margin.^24^ In earlier research, we found that similar DRS classification models are more prone to misclassifying *ex vivo* tumor locations with a higher tumor-margin distance and lower tumor area percentage.^65^ A similar trend can be seen in Fig. 5.

## 4 Discussion

In breast-conserving surgeries, it is essential to ensure negative resection margins to minimize the risk of local tumor recurrence and the potential need for subsequent surgeries or additional radiotherapy. However, no standard method is employed yet to support margin assessment by surgeons. With the ability to discriminate tumorous tissue from healthy tissue with high accuracy on the margin of *ex vivo* BCS specimens,^65^ DRS has the potential to be applied as a real-time margin assessment tool during breast-conserving surgeries. However, optimal strategies for selecting training data and the performance of different DRS classification models on breast tissue, which can subsequently be applied *in vivo* during BCS, has not been fully evaluated yet.

In this study, we therefore built an extensive dataset of DRS measurements on *ex vivo* breast tissue and *in vivo* breast tissue, which we used to develop four different types of classification models to evaluate the classification performance on DRS data acquired from *in vivo* breast tissue. We investigated which type of training data yielded optimum results for the classification model with the highest performance.

For all built model types, the classification performance when trained on DRS data acquired from *ex vivo* tissue was comparable to the classification performance when trained on DRS data acquired from *in vivo* tissue. This could be explained by the fact that all selected features were situated in the near-infrared wavelength range, where fat and water are the primary absorbers. In an earlier study of our group, it was found that the fat fraction in combination with the total volume of fat and water provided the best discrimination between tumorous breast tissue and healthy breast tissue.^55^ This optical parameter did not change significantly when comparing *ex vivo* tissue measurements with *in vivo* tissue measurements.^56^^,^^66^ On the other hand, the rapid change in ratio of oxygenated to deoxygenated hemoglobin post surgery substantially impacts the optical absorption in the visual wavelength range. Therefore, based on our previous results, no features were extracted from the visual wavelength range.

The RUSBoost has the highest classification performance on any type of training data compared with the other investigated models. The highest performance is achieved when the RUSBoost is trained on a combination of DRS data acquired from *ex vivo* tissue and *in vivo* tissue, with a MCC of 0.76. The explanation for the high performance of this particular model is that the RUSBoost model alleviates the imbalance of the *in vivo* dataset using random under sampling and boosting.^67^

When analyzing the three ROC curves for the RUSBoost models trained on DRS data from (1) *ex vivo* tissue, (2) *in vivo* tissue, and (3) *ex vivo* and *in vivo* tissue, it was evident that the AUCs of all models were comparably high. Similarly, all models had a high sensitivity and specificity at the optimal operating point. The sensitivity of all models is higher compared with the specificity, which is desirable because missing any tumor tissue on the margin has a more negative impact on the clinical outcome of a patient. Thus our data show that it is possible to mainly use *ex vivo* data for training the models. The results showed that training on a combination of *ex vivo* and *in vivo* data yields the highest performance metrics, with an AUC of 0.98, sensitivity of 96.7% (95% CI 95.6% to 98.2%), and specificity of 90.6% (95% CI 86.3% to 97.9%). A probable explanation for the slightly higher performance is the higher amount of available data for training compared with the models trained on only *ex vivo* or only *in vivo* data.

It should be noted that during this research, we carefully correlated the measured tissue locations to the corresponding region in the H&E sections. However, the area percentages of tissue types and the tumor-margin distances could only be calculated from the 2D H&E region derived from a few cell layers, whereas the actual probed volume is a bigger 3D volume. Therefore, the calculated parameters in the H&E section will not completely correspond with the probed tissue volume. Furthermore, it should be mentioned that the tissue percentage distribution among the *ex vivo* tissue locations is slightly different from the *in vivo* tissue locations. A possible explanation for this difference is that the data acquisition on *ex vivo* tissue could be performed in a more controlled method, and US imaging was available to localize tumor areas in some specimens. Because the data distribution is slightly different for the *in vivo* locations, including tumor locations with higher tumor percentages and healthy locations with higher fat percentages, this dataset could possibly lead to less misclassifications compared with the *ex vivo* dataset. This could partially explain the finding in a previous study in which we built an RUSBoost model on an *ex vivo* dataset to distinguish tumorous tissue from healthy tissue up to 2 mm from the margin *ex vivo* and found a sensitivity of 93% and a specificity of 75%, which were lower performance metrics compared with the findings in this study (sensitivity of 97% and specificity of 91%).^65^ An underexposed point in our manuscript would be the influence of different patient and tumor characteristics on the classification accuracy of the models. Unfortunately, the individual groups in our study were too small to perform any subgroup analyses. However, this would be an interesting point to study in the future.

Our ultimate goal is the clinical application of DRS for real-time margin assessment during BCS to improve surgical outcomes. For this purpose, it is important to perform the DRS measurements at the exact tissue locations with a high probability of a (focally) positive margin. There are two possible options to solve the issue of finding these locations: (1) using a second modality to find the measurement locations and (2) applying continuous DRS measurements. For the first option, US imaging combined with deep learning based tumor segmentation could be a valuable addition. In an earlier study, we found a median dice score of 0.88 when using an ensemble approach for tumor segmentation in US images of BCS specimens,^65^ which shows great potential to be applied as a modality to find the correct locations for margin assessment. In the second option, the DRS probe could be moved over the entire margin, permitting quick measurements and classification in a continuous manner. The feasibility and diagnostic accuracy of both options should be investigated in a follow-up study.

## 5 Conclusion

In this study, we have advanced toward the use of fiber-optic DRS for intra-operative margin assessment during BCS. We built an extensive set of DRS data acquired from *ex vivo* and *in vivo* breast tissue and built several classification models to discriminate tumorous tissue from healthy tissue up to 2 mm from the resection margin. The optimum classification model had a similarly high performance for classifying DRS data from *in vivo* tissue, when trained on *ex vivo* and/or *in vivo* data. By training on a combination of *ex vivo* and *in vivo* data, we achieved an MCC of 0.76, a sensitivity of 96.7% (95% CI 95.6% to 98.2%), a specificity of 90.6% (95% CI 86.3% to 97.9%), and an AUC of 0.98. These results show the potential of DRS to be applied as a tool to support margin assessment in real time during BCS. To achieve this ultimate goal, additional research needs to be performed regarding the optimum method to find the tissue locations with a high probability of a (focally) positive margin during BCS.

## Supplementary Material



## Data Availability

Data underlying the results presented in this paper are not publicly available at the time but may be obtained from the authors upon reasonable request.
