# Supplementary material for: Margin assessment during breast conserving surgery using diffuse reflectance spectroscopy
Source: J Biomed Opt. 2024 Apr 25;29(4):045006. doi: 10.1117/1.JBO.29.4.045006 (PMC11045169; doi:10.1117/1.JBO.29.4.045006)
Supplement: Supplementary file 1 [file JBO_029_045006_SD001.pdf]

Margin assessment during breast conserving surgery using diffuse reflectance spectroscopy

Dinusha Veluponnar[a,b,*], Lisanne L. de Boer[a,], Behdad Dashtbozorg[a,***], Lynn-Jade S. Jong[a,b], Freija Geldof[a,b], Marcos Da Silva Guimaraes[c], Henricus J.C.M. Sterenborg[d], Marie-Jeanne T.F.D. Vrancken-Peeters[a], Frederieke van Duijnhoven[a], Theo Ruers[a,b]**
[a]Netherlands Cancer Institute, Antoni van Leeuwenhoek, Department of Surgery, Image-Guided Surgery, Plesmanlaan 121, 1066 CX, Amsterdam, The Netherlands
[b]University of Twente, Faculty of Science and Technology, Department of Nanobiophysics, Drienerlolaan 5, 7522 NB, Enschede, The Netherlands
[c]Netherlands Cancer Institute, Antoni van Leeuwenhoek, Department of Pathology, Plesmanlaan 121, 1066 CX, Amsterdam, The Netherlands
[d]Amsterdam University Medical Center, Department of Biomedical Engineering and Physics, Meibergdreef 9, 1105 AZ, Amsterdam, The Netherlands

*, Dinusha Veluponnar d.veluponnar@nki.nl **, Lisanne de Boer l.d.boer@nki.nl ***, Behdad Dasthbozorg b.dasht.bozorg@nki.nl

1 Diffuse reflectance spectroscopy setup

A schematic representation of the diffuse reflectance spectroscopy setup is shown in Figure 1.

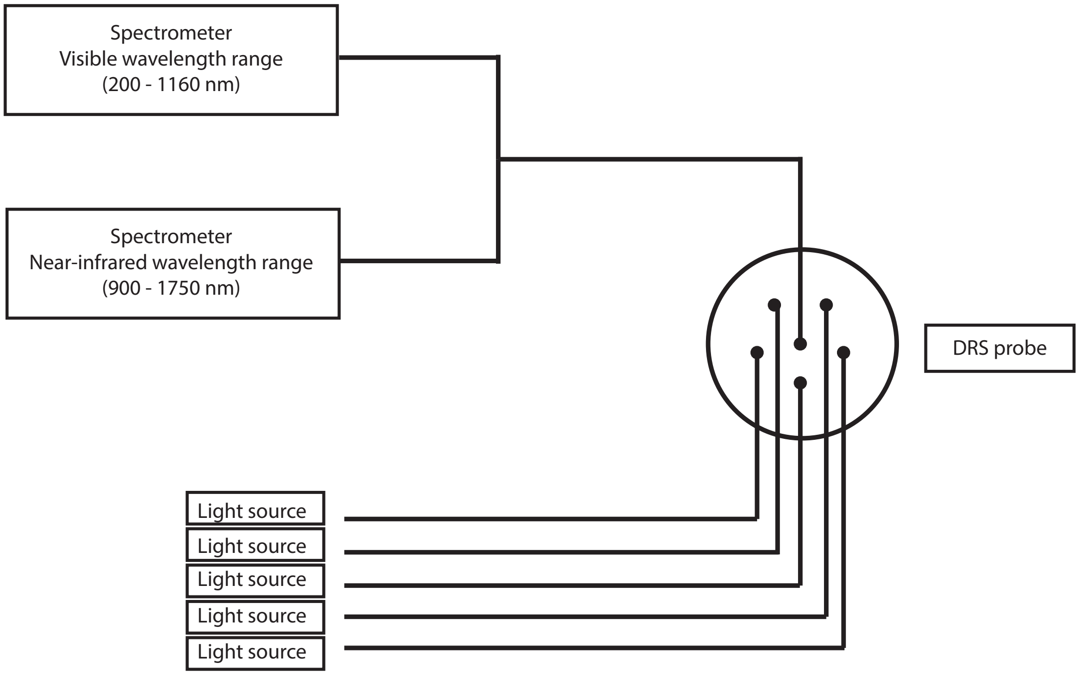

Fig 1: Optical measurements system. The diffuse reflectance spectroscopy setup consisted of a console with 5 identical light sources, coupled to two different spectrometers, and one in-house developed, handheld fiber optic DRS probe. The light sources were identical lamps with integrated shutters. Of both spectrometers, one covered a visible wavelength range from 200 to 1160 nm (Avantes, AVASPEC-HS2048XL-EVO), while the other covered a near-infrared wavelength range from 900 to 1750 nm (Avantes, AVASPECNIR256-1.7-RS).The DRS probe consisted of 5 peripheral illumination fibers placed in a circle around one receiving fiber in the center. Each light source was coupled to one illumination fiber. The source-detector fiber distance for all fibers was 2.0 mm.
